# Frictional Behavior and Tribofilm Formation of Organic Friction Modifiers under Severe Reciprocating Conditions

**DOI:** 10.1021/acs.langmuir.5c03784

**Published:** 2025-10-31

**Authors:** Marjan Homayoonfard, Sven L M Schroeder, Peter Dowding, Oliver Delamore, Ardian Morina

**Affiliations:** † Institute of Functional Surface, School of Mechanical Engineering, 4468 University of Leeds, Leeds LS2 9JT, U.K.; ‡ School of Chemical and Process Engineering, University of Leeds, Leeds LS2 9JT, U.K.; § Diamond Light Source, Harwell Science & Innovation Campus, Chilton, Didcot QX11 0DE, U.K.; ∥ Infineum UK Ltd., Milton Hill Business & Technology Centre, Milton Hill, Abingdon Ox13 6bb, U.K.

## Abstract

In pursuing environmentally friendly lubrication solutions, it is advantageous to employ organic additives that are free from heavy metals and have low or zero levels of phosphorus and sulfur functionalities. Organic friction modifiers strongly reduce friction and wear when added to engine oils through the formation of an adsorbed boundary film on the contacting surfaces. The mechanism of tribofilm formation and its chemical effects on friction reduction are not entirely understood. In this study, the lubrication mechanism of OFM was investigated with a new approach combining three different acylglycerols with varying ratios. The lubricating performance of mixtures of glycerol monooleate (GMO), glycerol trioleate (triolein), and glycerol dioleate (GDO), as well as individual GMO and triolein in PAO4, was evaluated under the boundary lubrication regime at two temperatures, 60 °C and 100 °C. A synergetic effect on tribological performance has been observed for the mixture formulation. This resulted in lower friction and wear than the single additive in the base oil at both temperatures. The HRTEM analysis indicated that the combination of different acylglycerols provides a thicker tribofilm compared to the single additive. The ToF-SIMS and NEXAFS analyses of the resulting tribofilms showed that at a temperature of 60 °C, the main components of the tribofilm were compounds formed by GMO decomposition and oleate ions, indicating that chemisorption plays a significant role in reducing friction at lower temperatures for the tested OFM additives.

## Graphical abstract

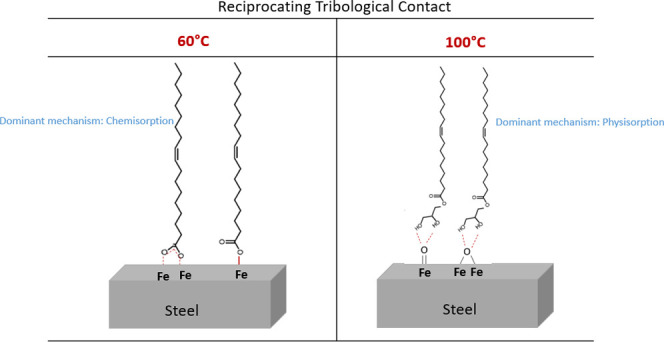

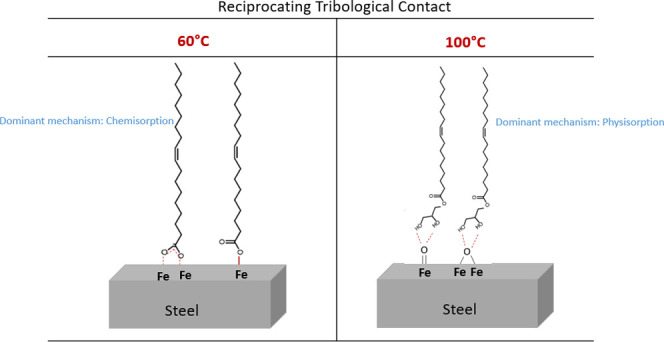

## 1 Introduction

In engine systems, friction and wear are principal causes of energy inefficiency and material degradation. Optimizing friction at tribological contacts is a vital and economical approach to enhance energy efficiency and prolong the service life of engine components. Boundary lubrication poses a significant challenge in engineering applications, as asperity-to-asperity contact becomes unavoidable, resulting in elevated friction and wear. To mitigate these effects, various lubricant additives are employed. The performance and efficiency of an engine are significantly influenced by the properties of the lubricant and the effectiveness of the lubrication system employed. Friction modifiers (FMs) are widely used among these additives due to their outstanding antiwear properties and their ability to enhance lubricity and energy efficiency, particularly under boundary lubrication conditions.^1,2^ Lubricant formulations used in automotive applications often include friction modifiers, such as organomolybdenum compounds and organic friction modifiers (OFMs). Organomolybdenum compounds, which typically contain heavy metals such as molybdenum, can lead to the discharge of hazardous emissions containing sulfated ash. In addition, they also contain sulfur, which is shown to impact the efficiency of exhaust gas recirculation systems. As a result, with stricter environmental regulations, the use of OFMs, composed mainly of carbon, hydrogen, and oxygen, has gained traction as a more environmentally friendly.^1,3–8^ OFMs are typically alkyl chain surfactants with a hydrophilic headgroup.^1,9–11^ Carboxylic acid, amine, ester, and alcohol are a few OFMs commercially used as lubricant additives.^9,12^ These friction modifiers minimize friction at asperity contacts through the formation of protective films. The performance of an OFM is primarily governed by its interfacial interaction and chemical reactivity with the surface.^13^

Esters have been widely used as an effective friction modifier due to their unique chemical and physical properties.^14,15^ Furthermore, their inherent polarity enhances their ability to adsorb strongly onto metal surfaces, thereby enabling the formation of protective boundary films that mitigate friction and wear under boundary lubrication conditions. Glycerol monooleate (GMO) is a monoester derived from fatty acids and is among the most widely utilized organic friction modifiers. As illustrated in Figure 1, its molecular structure consists of a glyceryl moiety linked to an oleyl chain through an ester bond.^16–18^ The positive effect of GMO in protecting the surface and reducing friction is extensively reported in literature, as it significantly improves tribological performance by forming protective films on the surface, however, the exact mechanism by which GMO reduces friction is still not fully understood. Different mechanisms for reducing friction seem possible based on the chemistry and structure of GMOs. The primary reaction involves one or both alcohol groups in the GMO forming a hydrogen bond with the oxygen on the iron oxide surface, enabling the GMO to create a boundary film either as an intact molecule or through interaction of the ester carbonyl oxygen with the iron on the surface.^19^ The second mechanism can involve the hydrolysis of GMO to oleic acid and glycerol, where only the oleate molecules contribute to the tribofilm formation. The third potential mechanism is that the GMO hydrolysis to oleic acid and glycerol, with both components contributing to the lubrication of the surface. The last and the least likely mechanism is the dissociation of GMO to oleic acid and glycerol, with only glycerol involved in the film formation.

**Figure 1:**
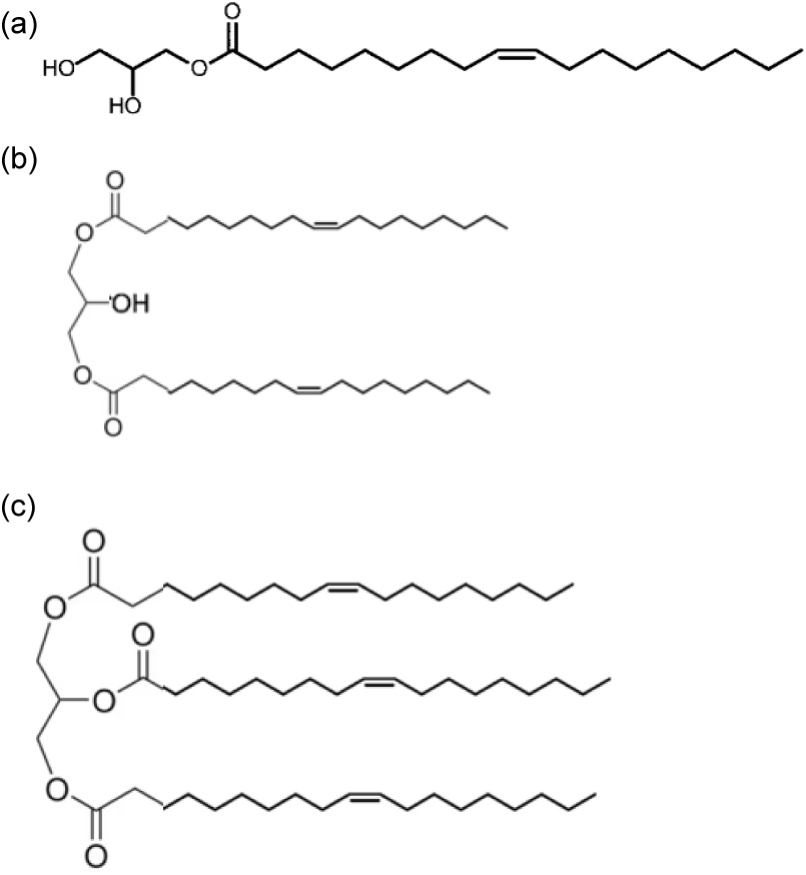
Molecular structures of (a) GMO, (b) glycerol dioleate, and (c) triolein.

The nature of the adsorbed GMO layer is dependent on the surface characteristics. Some researchers suggested that GMO lubricates steel surfaces by hydrolyzing into oleic acid, which then adsorbs onto the surfaces to prevent direct metal-to-metal contact,^9,12,17,20,21^ according to the Bowden–Tabor concept.^22^ This process is similar to previous methods in which fatty acids produced by the hydrolysis of esters with trace amounts of water were used to lubricate ferrous surfaces.^12^ In contrast, it has been proposed that GMO adsorbs onto the metal surface via interactions between its polar headgroup and oxygen atoms present in the surface oxide layer, leading to hydrogen bond formation.^19^ Topoloveck et al. suggested that GMO interacts with diamond-like carbon (DLC) surfaces in its original form.^23^ Wang et al. claimed that GMO friction reduction mechanism is dependent on the temperature.^24^ They suggested that at temperatures above 110 °C, chemisorbed films play a dominant role, whereas at lower temperatures, friction reduction is primarily achieved through physical adsorption of GMO on the surface. Despite extensive research, the relationship between the formation of reaction films by organic friction modifiers—particularly GMO—and their effectiveness in reducing friction remains inadequately understood.

This study experimentally evaluated the effectiveness of combining various organic friction modifiers, including GMO, mixed GO, and triolein, in reducing friction and wear under severe boundary lubrication conditions. The experiments are conducted under reciprocating motion at two different temperatures. These temperatures were chosen due to their relevance to automotive engine operating conditions. Post-tribological analysis of the decomposition products on the surface was carried out using Time-of-Flight Secondary Ion Mass Spectrometry (ToF-SIMS), Near-Edge X-ray Absorption Fine Structure (NEXAFS), and Scanning Electron Microscopy (SEM). Additionally, the film thickness was measured by higher-resolution transmission electron microscopy (HRTEM).

## 2 Materials

Glycerol monooleate (GMO), glycerol trioleate (triolein), and oleic acid with ≥99% purity were supplied from Sigma-Aldrich. PAO4 base oil (viscosity index 124, flash point 222 °C, pour point −69 °C) and mixed GO, which contains 50% GMO, approximately 40% glycerol dioleate (GDO), and over 10% triolein, were provided by Infineum. The molecular structures of all employed OFMs are presented in Figure 1. The OFMs were used as received. The friction of oleic acid was investigated to determine if GMO reduces friction through hydrolysis to oleic acid and glycerol. Test solutions were prepared by dissolving 1 wt % of the respective additive in the base oil, followed by heating to 60 °C and continuous magnetic stirring to ensure complete homogenization. All tribological experiments were performed immediately after preparing the sample.

## 3 Test Method

The frictional performance of the lubricants on the steel plate has been evaluated using a TE77 reciprocating tribometer (Figure 2). Since the reciprocating motion is prevalent in many engineering systems, such as reciprocating pumps and internal combustion engines, the TE77 tribometer was chosen for this study.^25^ Material properties and dimensions of specimens are detailed in Table 1. Tests were carried out for 2 h at a sliding speed of 0.02 m s^–1^ under a normal load of 40 N, corresponding to an average contact pressure of approximately 1 GPa. Maximum contact pressure was calculated using Hertzian contact theory. The oscillation frequency was set to 4 Hz with a stock length of 5.0 mm.

**Figure 2:**
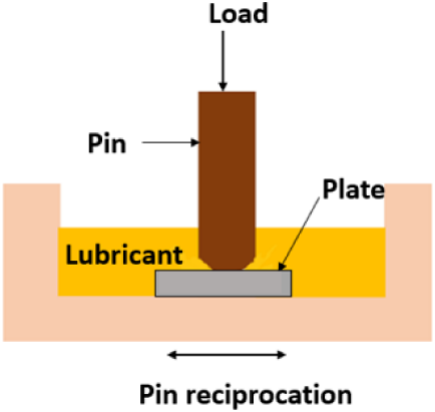
Schematic image of TE77 setup.

The White Light Interferometry, NPFLEX 3D Surface Metrology System from Bruker, was used to evaluate the wear scar diameter of the pins after the tribological test. Before the measurement, the steel surfaces were cleaned with heptane to eliminate any residual oil. 3D images of the worn scar were taken, and the resulting data were processed and analyzed using Bruker’s Vision 64 software. A diameter value was obtained for each wear scar by averaging two perpendicular measurements of the circular scar. The wear volume loss of the pin was calculated by using the following equations.^26^

1 $$v_{\mathrm{pin volume loss}}=\frac{\pi h^{2}\left(3R-h\right)}{3}$$

2 $$h=R-{\left(R^{2}-r^{2}\right)}^{0\cdot 5}$$

where *h* is spherical cap height (μm), *R* is sphere radius (μm), and *r* is wear scar radius (μm).

The plate specimens were analyzed by SEM (Carl Zeiss AG) to identify the wear mechanism. To better investigate the underlying wear mechanisms, the worn surfaces were treated with an EDTA solution to remove the tribofilm before conducting the analysis.^27^ The EDX instrument from Oxford was subsequently employed to determine the chemical elements’ distribution.

**Table 1:** Material Properties of Specimens

	Pin	Plate
Material	Steel EN31	Steel EN31
Elastic modulus (GPa)	207	207
Poisson’s ratio	0.3	0.3
Roughness (nm)	35–50	400–600
Dimensions (mm)	Hemispherical end radius, 10 radius	7 × 7 × 3

## 4 Surface Chemical Analysis

Post-tribological characterization of the additive-derived films on the steel surface was carried out using High-Resolution Transmission Electron Microscopy (HRTEM). The combination of Energy Dispersive X-ray Spectroscopy (EDX) and High-Angle Annular Dark-Field (HAADF) imaging facilitated a comprehensive evaluation of the elemental distribution and chemical composition. The cross-sections of the worn surfaces were prepared for TEM observation using advanced High-Resolution Monochromated Focused Ion Beam (FIB) technology.

Time-of-Flight Secondary Ion Mass Spectrometry IV (ToF-SIMS, ION-TOF GmbH, Münster, Germany) was employed to study the chemical composition of the tribofilm. ToF-SIMS is a technique capable of analyzing thin films and providing information about organic chemical structures.^10,28^

A bismuth liquid metal ion gun (Bi_3_^+^) operated at 25 kV with a pulsed target current of approximately 1 pA was used as the primary ion source. Surface analysis was conducted on the wear tracks of the steel plates. Data for each specimen were collected from three distinct regions, each measuring 500 × 500 μm^2^, at an image resolution of 256 × 256 pixels. The analyzed mass range (*m*/*z*) spanned from 1 to 410. Positive and negative ion spectra were obtained from the wear tracks. For consistency and comparative purposes, selected spectra were normalized by dividing the ion intensity of interest by the total ion intensity, and identical *Y*-axis scales were applied.

Near-Edge X-ray Absorption Fine Structure (NEXAFS) spectroscopy was used as a complementary surface analysis technique. Information regarding the chemical composition, functional groups, and orientation of surface-adsorbed molecules can be obtained through NEXAFS spectroscopy.^29^ NEXAFS spectra were acquired inside and outside the worn tracks on the used steel plates. NEXAFS characterization was performed at Diamond Light Source beamline VerSoX B07–B, utilizing the near ambient pressure NEXAFS end station enabling fast sample transfer.^30^ A notable advantage of this end station is its capacity for analysis of real-world samples under ambient, rather than ultrahigh vacuum, conditions. This facilitates ex situ investigations of samples that are otherwise not compatible with ultrahigh vacuum environments.^34^ Measurements were carried out using total electron yield detection, measuring the drain current through the sample mounting plate with the soft X-ray beam at normal incidence and room temperature. Each sample was affixed to a copper foil using carbon tape. To correct for beamline-induced shifts and surface charging, the photon energy scale was calibrated by introducing a trace amount of nitrogen gas into the sample chamber. The sharp absorption peak of nitrogen at 400.8 eV served as the reference point for calibration. The sample chamber was maintained under 1 mbar of Helium.He was selected for its lack of absorption features near the C, N, and O K-edges and its ability to reduce interference from fluorescence-related gas-phase absorption in the electron yield signal. The incident X-ray intensity (*I*_0_), necessary for calculating the absorption spectra, was obtained by measuring the helium gas-phase spectrum after removing the sample holder from the beam path. The details of the spectrum normalization process with this gas phase I0 spectrum have been documented in detail in a previous study.^31^

## 5 Results and Discussion

### 5.1 Frictional Performance

Figure 3 shows the friction coefficient of the tested lubricants over time at two different temperatures, 60 and 100 °C. The results show that PAO4 exhibits the highest friction at both temperatures, compared to each solution containing organic friction modifiers. A marked increase in friction was observed for the base oil with prolonged rubbing, regardless of the operating temperature. This increase is primarily due to accelerated wear on the pin and plate surface. GMO, mixed GO, and oleic acid demonstrate very similar behavior. Throughout the test, their coefficient of friction remains low and stable. This result indicates that the adsorption of these additives effectively reduces friction between the contact surfaces. Mixed GO showed the lowest friction, and triolein exhibited the greatest friction coefficient among all tested samples at both temperatures. The higher friction of triolein could be attributed to its structure. The lower adsorption of triolein on the steel surface might result from higher steric hindrance. Triolein has a large molecule that can prevent reaction with the surface. Additionally, the findings from molecular dynamics simulations indicated that intermolecular hydrogen bonding has a crucial role in stabilizing the film monolayers.^32^ GMO has two and GDO has one free hydroxyl group, respectively, while triolein does not have any. At the start of rubbing, OFM molecules randomly adsorb on the metal surface.^33^ While GMO and GDO molecules are capable of forming hydrogen bonds with surface hydroxyl groups, the absence of hydroxyl moieties in triolein prevents such intermolecular interactions, leading to weaker surface affinity.^19,34^ Extended rubbing can result in the decomposition of these additives, forming oleic acid. The comparable friction coefficient of mixed GO to oleic acid supports this assertion.^35,36^ The significant reduction in the coefficient of friction observed with the mixed GO formulation is attributed to the synergistic effect of the combined lubricants.

**Figure 3:**
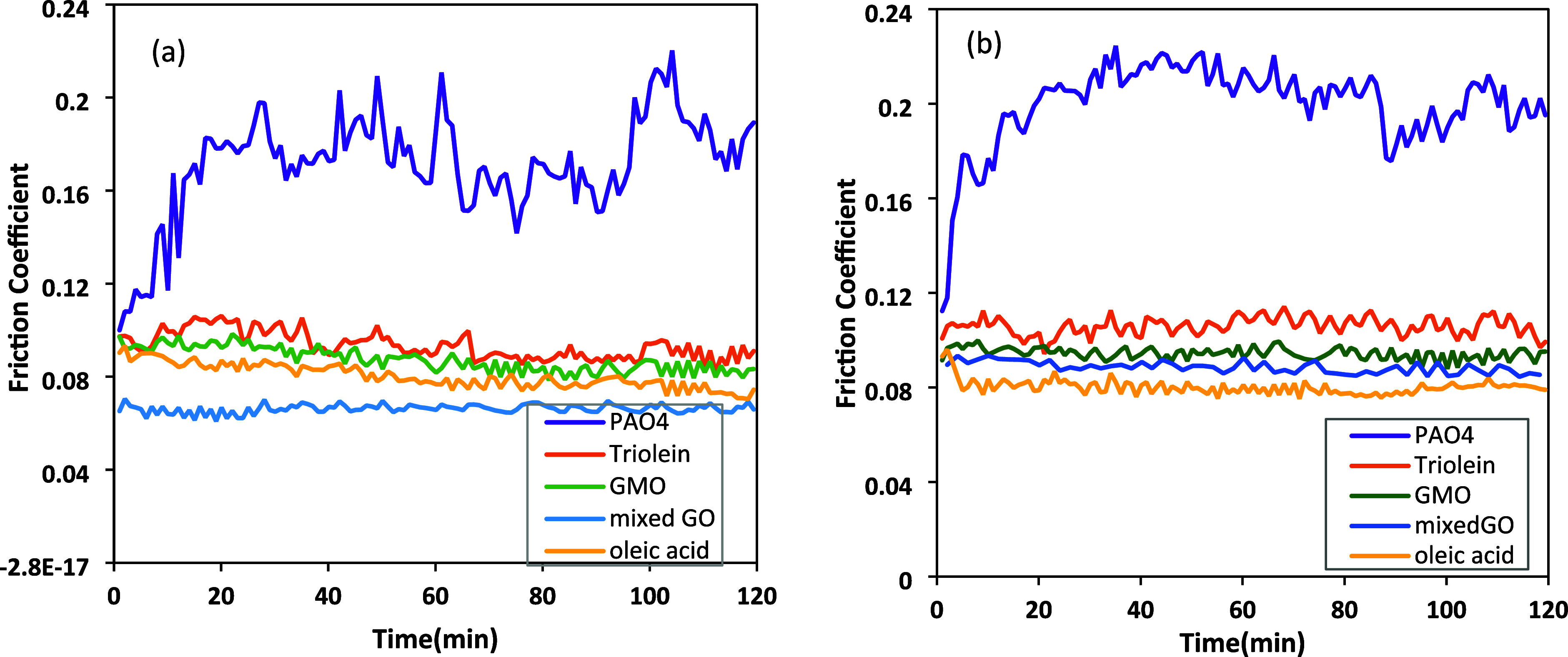
Coefficient of friction vs time at (a) 60 °C and (b) 100 °C.

The mean friction against time is plotted in Figure 4. The mean friction coefficients were calculated by averaging COF values over the final 20 min of the test, representing the steady state phase.

**Figure 4:**
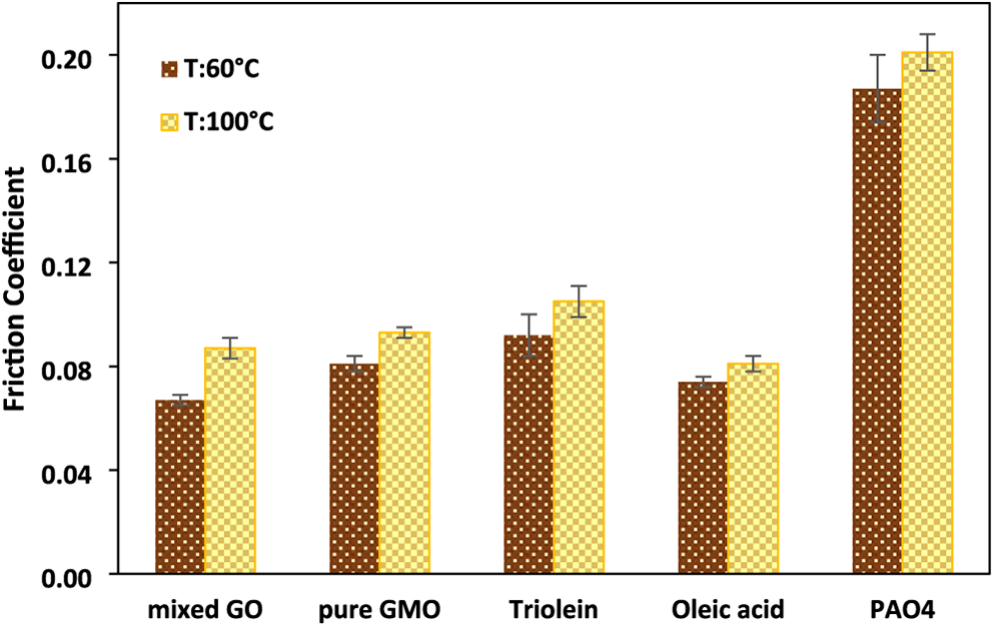
Mean coefficient for the last 20 min of tribotest for different lubricants in PAO4 at (a) 60 °C and (b) 100 °C.

The results show that all additives exhibited lower friction at a lower temperature. This phenomenon is likely due to the formation of a durable tribofilm that cannot be removed easily from the surface. While previous studies suggested that physical adsorption is the primary mechanism for reducing friction at lower temperatures^24^ and higher temperatures leading to the thermal decomposition of these additives,^34^ the current study indicates that chemisorption could also have a role in reducing friction at lower temperatures. This finding suggests that the conditions encountered during rubbing at a temperature of 60 °C promote the decomposition process of GMO, consistent with previous reports.^9,17,37^ The lower friction observed at 60 °C may result from the formation of a thicker and more stable tribofilm. Sections 5.3 and 5.4 will show the TEM and ToF-SIMS analysis of the film formed at this temperature, respectively. This observation supports earlier studies indicating that thicker boundary films are more effective in minimizing asperity–asperity interactions.^38^ In contrast, the higher friction observed at 100 °C may result from the desorption of tribofilm from the surface. As temperature increases, the thermal agitation of molecules also rises, eventually reaching a point where the molecular orientation is disrupted, and adsorption fades entirely.^24,39^ These results suggest that 100 °C may represent the transition temperature for these additives on an iron oxide surface under reciprocating motion. Overall, the findings highlight the significant influence of temperature on the frictional behavior of organic friction modifiers in alignment with previous studies.^24,40^

### 5.2 Wear Mechanism

The average wear volume of the pin after 2 h of sliding test in various organic friction modifiers was analyzed by white light interferometry (WLI) and an optical microscope to understand the antiwear characteristics of these lubricants. The wear volume results are presented in Figure 5. All lubricants showed lower volume loss compared to the base oil at both temperatures. A comparison of the wear volume indicates that tribofilms formed from additives effectively reduce wear, except for triolein at a temperature of 100 °C. With increasing temperature, the wear volume of the base oil lubricated pin increased. This increase can be attributed to the low viscosity of PAO4 base oil, which intensifies asperity contact under harsher lubrication conditions, resulting in greater wear.^41^

**Figure 5:**
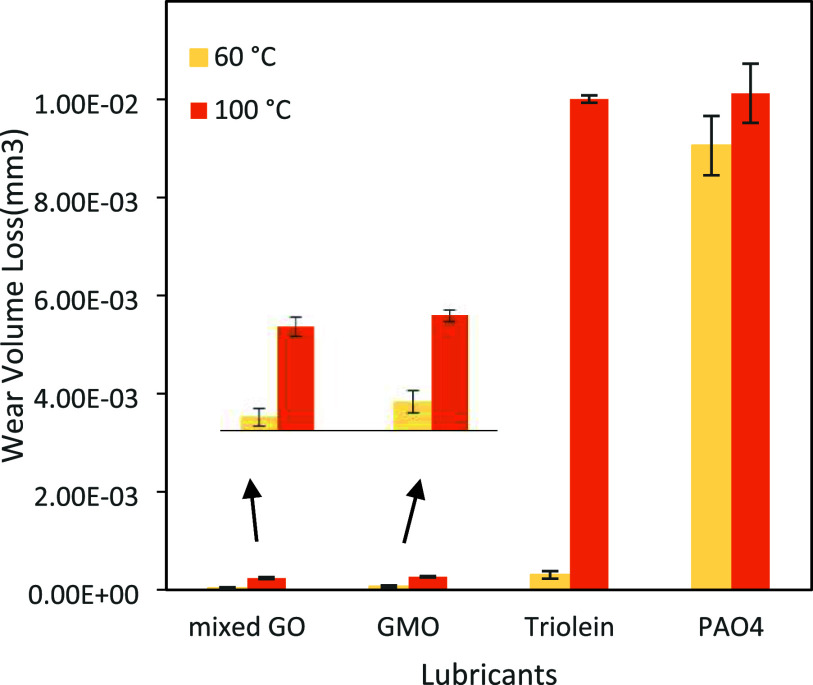
Average of wear volume from TE77 pins.

Notably, the wear volume loss of the pin was significantly lower for all additives at the lower temperature, which likely due to the formation of more durable tribofilm on the contacting surfaces, acting as the protective barrier. This observation has been confirmed by ToF-SIMS analysis as discussed in detail in Section 5.4. Among the tested additives, the mixed GO exhibited the lowest wear volume loss, indicating a synergistic effect resulting from the combination of these additives. Figure 6 presents the optical microscope images of pin surfaces tested with various lubricants at 60 °C. A clear difference in wear severity was noted among the different tested lubricants. A polishing effect was evident on the pin surface lubricated with GMO and mixed GO. Additionally, the lowest average wear scar diameter was observed for the mixed GO pin surface. These results indicate that the combination of these three oleates improved the wear compared to the individual additives. This enhancement is further supported by plate wear scar analysis through SEM analysis in Figure 7. The wear tracks on the plates were analyzed with SEM-EDX to assess the wear mechanisms.

**Figure 6:**
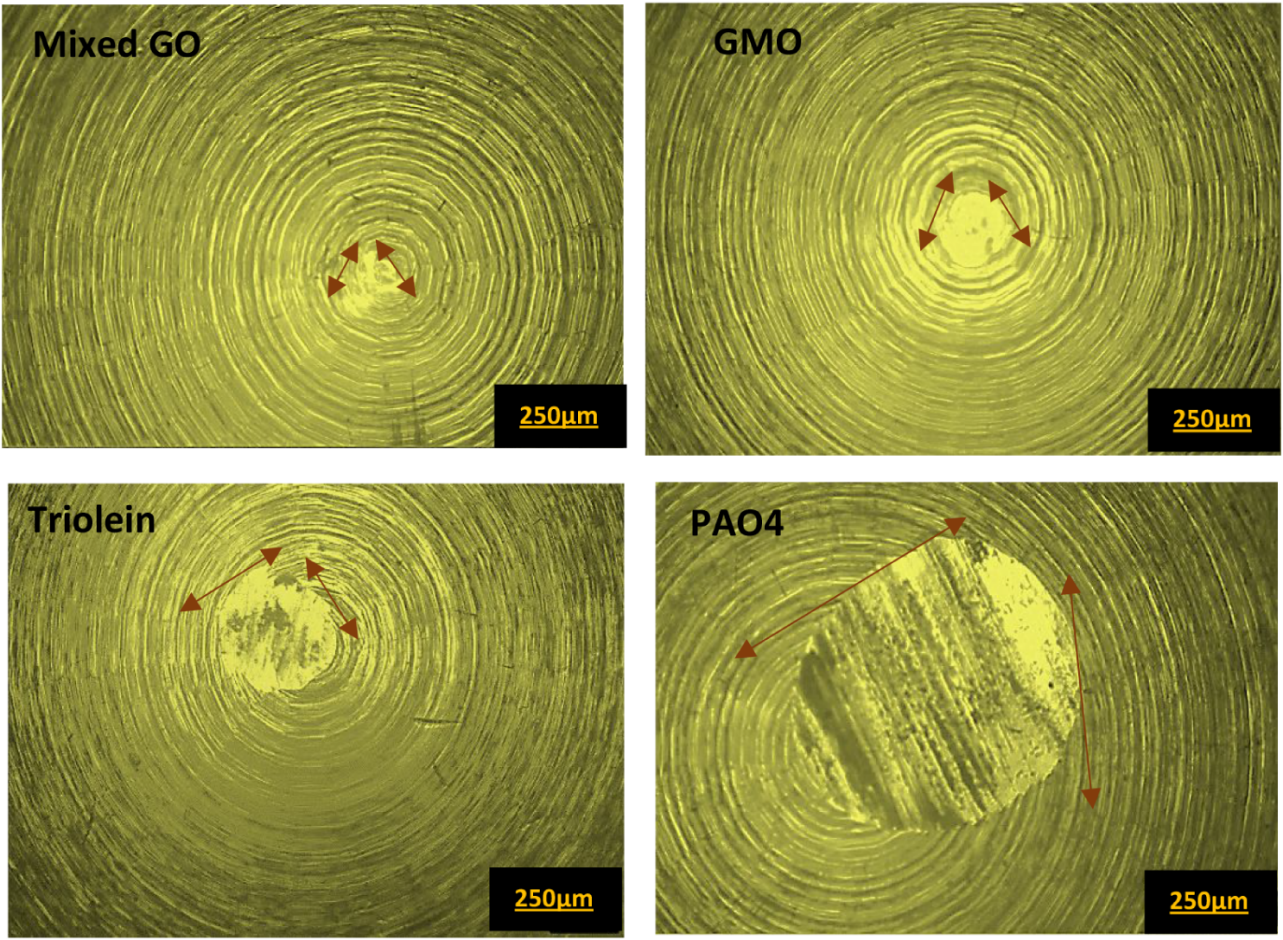
Optical microscope images of pin wear scar, following tests at 60 °C.

**Figure 7:**
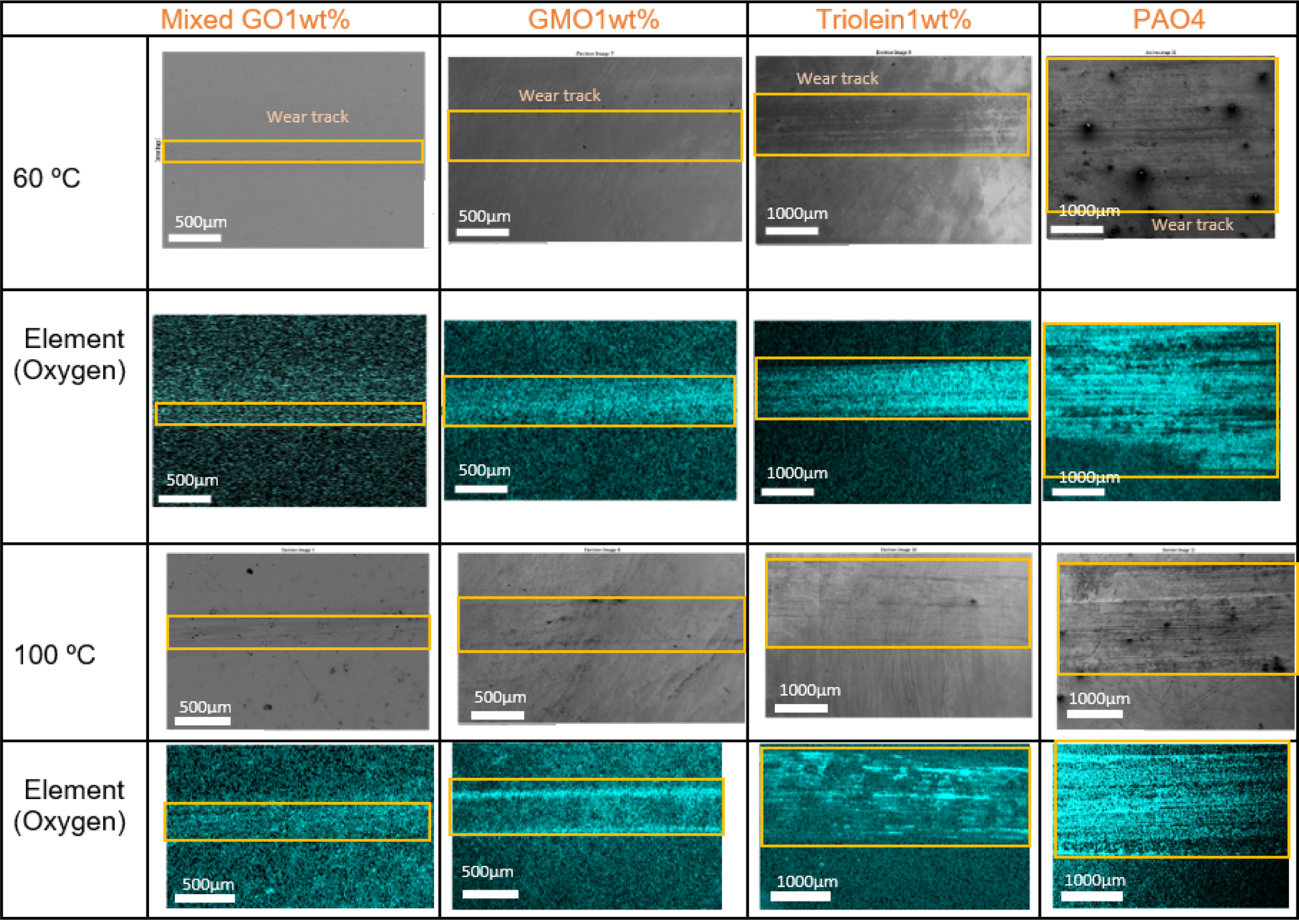
SEM-EDX analysis of the plate worn surface following test with various lubricants at 60 and 100 °C.

Based on the SEM images shown in Figure 7, the plate tested with PAO4 exhibited deep grooves, indicative of abrasive wear. In contrast, the plates lubricated with GMO and mixed GO displayed a more polished and uniform surface, indicating improved surface protection. The surface morphology of the triolein-lubricated plate revealed slight evidence of abrasive wear and a heterogeneous topography, suggesting less effective tribofilm formation compared to the other oleate-based lubricants.

SEM images revealed that smaller wear scars developed on the contacting surfaces at lower temperatures compared to those observed at higher temperatures. Among the tested additives, triolein produced the largest wear tracks at both temperatures, which can be attributed to its molecular structure. As previously discussed, the significant steric hindrance associated with its bulky molecular configuration limits its ability to adsorb effectively onto the surface. Furthermore, the absence of hydroxyl groups in triolein hinders the formation of strong, adherent films, resulting in tribofilms with low shear strength that are more prone to removal under severe operating conditions. At both temperatures, the GMO produced wider wear scars on the plate than the mixed GO. As shown by the HRTEM results discussed in the following section, the tribofilm formed by the mixed GO solution is thicker than that formed by GMO. Thicker films are known to exhibit greater durability and improved resistance to removal under severe loading conditions.^40^

A greater oxygen concentration in the wear track was found by EDX examination of the plate surface (Figure 7), suggesting a highly oxidized surface under tribological load,^42^ particularly for PAO4 and triolein at both temperatures. Our results also suggest that the maximum coefficient of friction of the additives correlates with an increase in wear track width. This observation aligns with previous studies, which have shown that thicker organic friction modifier layers produce narrower wear tracks, whereas thinner OFM layers lead to wider tracks.^16^

### 5.3 Tribofilm Thickness

The thickness of tribofilm in a lubricated system reflects the effectiveness of lubricant additives. It is the key factor for assessing the surface protection and friction reduction capacity of organic friction modifiers. In this study, HRTEM was employed to quantify a thickness of tribofilms generated on the plate surface lubricated by GMO and mixed GO, following the experiment at 60 °C. As the mixed GO exhibited the lowest friction at this temperature among all tested additives, tribofilm thickness was measured for this formulation and GMO to investigate whether film thickness contributed to the observed reduction in friction. Figure 8 presents the TEM images from FIB lamellae. Cross-sectional analysis of the tribofilms revealed a clear difference in film thickness between the two lubricants. A thick tribofilm with an average thickness of 108 nm was formed after 2 h of sliding under mixed GO lubrication, as seen in Figure 8a. The GMO tribofilm was significantly thinner, approximately one-third of the mixed GO tribofilm thickness (Figure 8b). The iron oxide layer was notably thicker on the surface tested with GMO, suggesting that this additive promoted greater surface oxidation than the mixed GO, as shown in Figure 8b.

**Figure 8:**
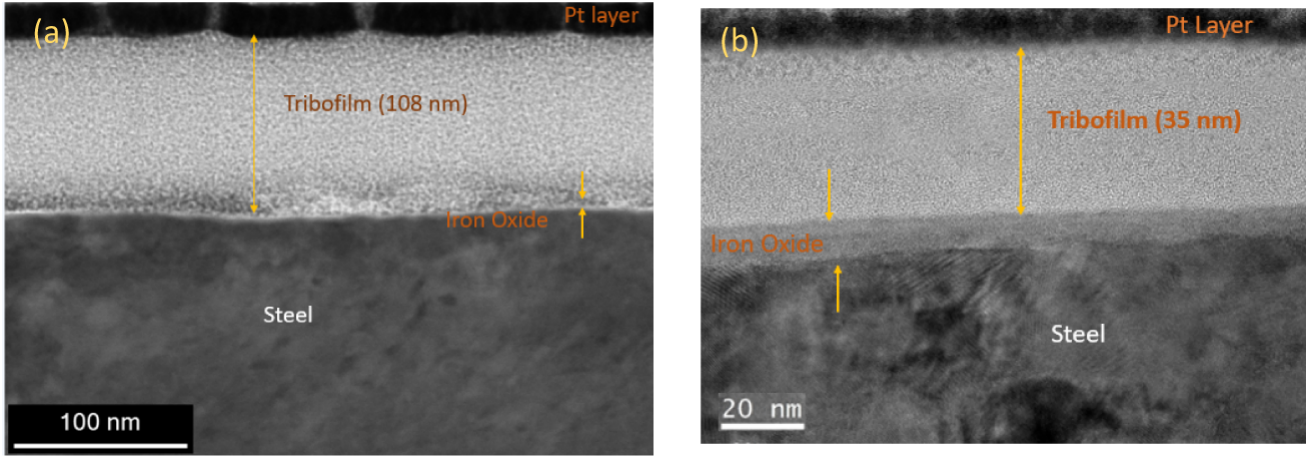
HRTEM images for the formed tribofilm at 60 °C for (a) mixed GO and (b) GMO.

These findings reveal that the chemistry of the organic friction modifiers significantly influences the thickness of the surface reaction layer, thereby impacting tribological performance. This observation is consistent with the previous study.^43^ In addition, high contact pressure facilitates film growth.^34,40^ Mixed GO contains GMO, di- and tri substituted glycerol, and these molecules decompose at harsh contact conditions and produce more oleate molecules. The formation of chemisorbed multilayer tribofilms on the contact surface is promoted by the presence of iron oleate ions, which play a key role in reducing friction.

The cross-sectional structure and elemental distribution of the tribofilm were investigated by EDX. The EDX mapping and high-angle annular dark field (HAADF) are illustrated in Figure 9. The tribofilm consists of C-enriched layers. The significant amount of C within the wear tracks can be attributed to the adsorption of OFM on the surface.^44^ As shown in Figure 9b, the markedly higher oxygen concentration observed for the GMO sample suggests the formation of a relatively thick oxide layer.

**Figure 9:**
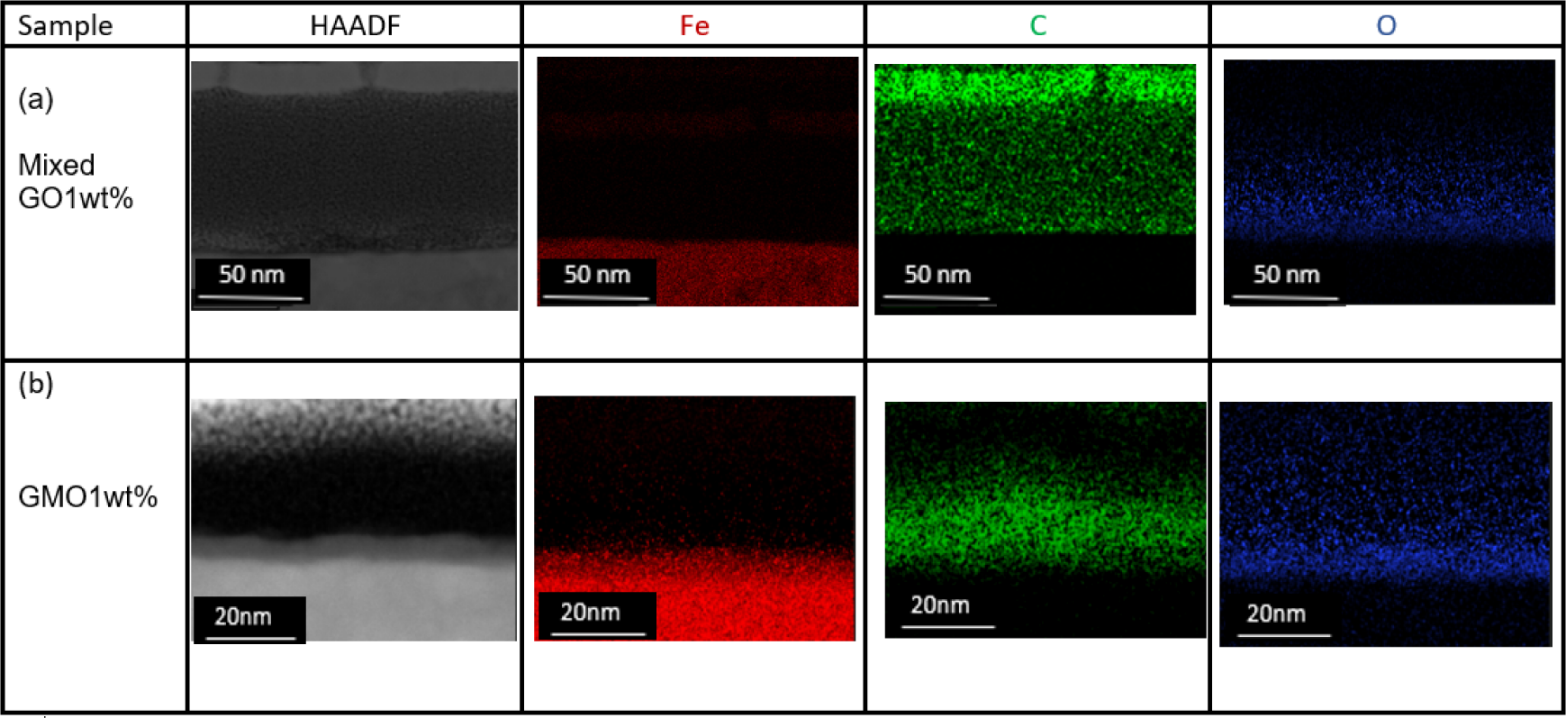
HAADF image and EDX measurement by HRTEM for (a) mixed GO and (b) GMO tribofilm at a temperature of 60 °C.

### 5.4 Tribofilm Chemistry

To understand the friction reduction mechanism of GMO, mixed GO, and triolein under a boundary lubrication regime at 60 °C and 100 °C, ToF-SIMS analysis was conducted to obtain the chemical composition of the formed tribofilms. It is proposed that the GMO undergoes hydrolysis to oleic acid and glycerol, and the chemisorbed iron oleate film is formed from the cleavage of the oleic acid, resulting in friction reduction. Previous studies have proposed that the presence of ions corresponding to [C_17_H_33_COO]^−^ indicates the chemical adsorption of GMO on the surface.^36,45^ Additionally, ion fragments such as [C_21_H_39_O_3_]^−^, [C_17_H_33_O_3_]^−^, [C_18_H_33_O]^−^, [C_16_H_31_O]^−^, and [C_16_H_29_O]^−^ can be attributed to GMO decomposition.^36,45–47^ These ions result from the interaction between the cleaved alkyl chains of GMO and oxygen present on the iron oxide surface. In this study, the key objective of this analysis was to determine whether any of these ions could be identified. Figure 10 shows the initial fragment ions resulting from the decomposition of GMO.

**Figure 10:**
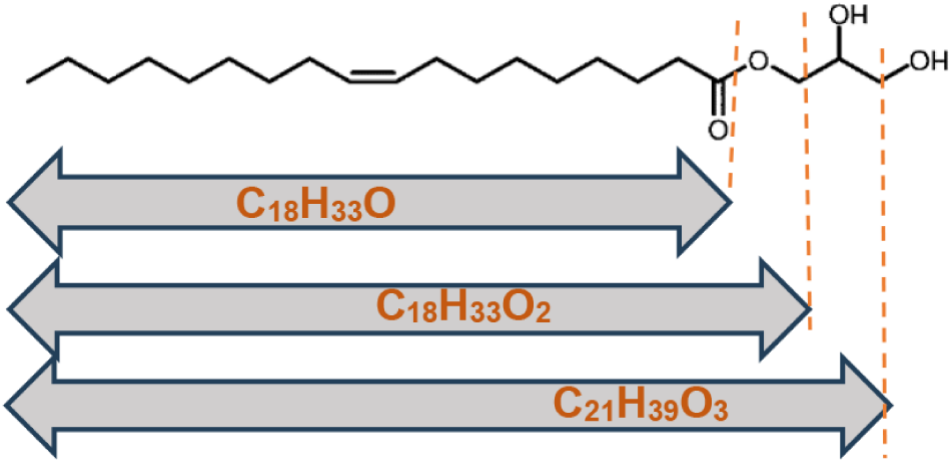
Initial fragment ions resulting from GMO decomposition.

The negative ion spectra within the mass range of 220 to 320 u, acquired from regions inside the wear tracks on steel plates tested with GMO, a mixed GO and triolein, at 60 °C and 100 °C, are presented in Figure 11. Spectra were collected at three distinct positions along the wear track to determine whether the chemical composition of the surface varied under frictional conditions. The characteristic fingerprint peak of the oleate ion ([C18H_33_O_2_]^−^) at 281.2 u was detected for all additives at 60 °C. However, this ion was no longer observed at 100 °C. These findings indicate that chemisorption is a key mechanism contributing to friction reduction at lower temperatures.

**Figure 11:**
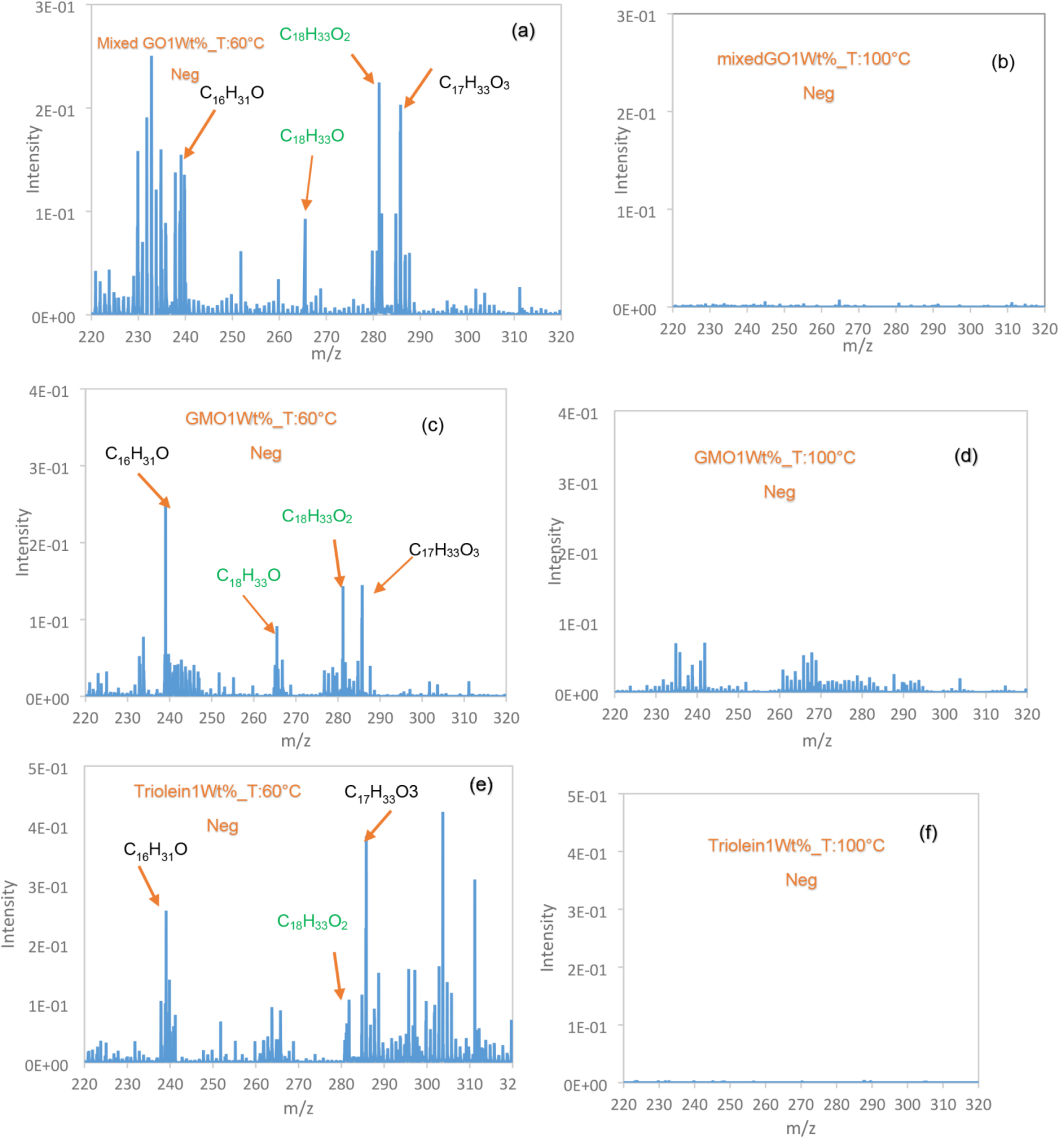
Negative ions from ToF-SIMS analysis of inside the wear scar generated with (a) mixed GO at 60 °C, (b) at 100 °C, (c) GMO at 60 °C, (d) 100°, (e) triolein at 60 °C, and (f) 100 °C.

The appearance of the oleate ion ([C_18_H_33_O_2_]^−^) at 60 °C can be rationalized by considering the relative bond dissociation energies (BDEs) within the GMO molecule. GMO contains several types of bonds with distinct bond strengths. Among all bonds present in the molecule, C–O (ester), O–H (hydroxyl), C–C, C–H, and C=O, the ester C–O bond exhibits the lowest bond dissociation energy, approximately 350–380 kJ mol^–1^, making it the weakest bond in the molecule.

At 60 °C, the applied contact pressure and shear during sliding generate localized asperity flash temperatures that are significantly higher than the bulk temperature. These local conditions can supply sufficient energy to overcome the relatively low BDE of the ester C–O bond (∼350 kJ mol^–1^),^48^ promoting partial hydrolysis or cleavage of GMO into oleic acid and glycerol. In contrast, stronger bonds such as O–H or C=O remain largely intact at this temperature.

During the initial stage of rubbing, GMO and GDO adsorb onto the metal surface through one or more of their glyceryl hydroxyls, forming hydrogen bonds with the ferrous substrate.^19,34^ Continued rubbing under high contact pressure leads to a rise in contact temperature due to frictional heating. As a result, the decomposition of these FMs to oleic acid and glycerol may occur at asperity–asperity contact, where localized temperature is substantially greater.^28,49^ Oleate ions subsequently form strong chemisorbed bonds with the surface, whereas glycerol interacts predominantly via hydrogen bonding. However, because hydrogen bonds are comparatively weaker, glycerol gradually desorbs from the surface as the contact temperature increases. A previous study claimed that with increasing temperature, the thermal motion of the molecules also increases until the temperature reaches the point that the molecules become disoriented and eventually desorb from the surface.^33^ As a result, it can be suggested that the rise in temperature in the test 100 °C leads to the desorption of the molecules from the surface, explaining the higher friction observed at this temperature. These findings indicate that sliding motion plays a vital role in both the formation and stability of tribofilms. At boundary lubrication conditions, sliding not only facilitates the mechanochemical activation of organic friction modifiers, promoting their decomposition into reactive fragments, but also supports the growth of chemisorbed tribofilms. At lower temperatures, this contributes positively to surface protection. However, under higher thermal and mechanical stress, sliding may also accelerate the removal of weakly adsorbed molecules, reducing tribofilm effectiveness. The combined influence of temperature and mechanical shear is therefore essential in determining tribofilm behavior and overall tribological performance.

A higher intensity peak of oleate ions [C_18_H_33_O_2_]^−^ fragment was observed for mixed GO compared to the two other additives. As previously mentioned, mixed GO contain three different glycerol oleates differing in the number of oleyl groups: GMO (one), GDO (two), and triolein (three). Under severe contact conditions, each of these additives is susceptible to hydrolysis, resulting in the generation of oleate ions. Consequently, the mixed formulation yields a higher overall concentration of oleate ions, which accounts for the more intense signal observed in the ToF-SIMS spectra.

The ion fragment associated with additive decomposition was also detected at *m*/*z* 265 in the negative ion spectra of mixed GO, GMO, and triolein solutions at the lower temperature. As shown in Figure 11a,c,e, the ion fragments [C_16_H_31_O] and [C_17_H_33_O_3_], corresponding to *m*/*z* 239 and 285, respectively, were detected inside the wear tracks following the tests conducted at 60 °C.

Moreover, the higher concentration of hydrocarbon fragments with the general formula [C_n_H_m_]^+^ observed within the wear track provides evidence that GMO and other additives underwent decomposition under the applied tribological conditions.^40^

Based on the tribological results, where friction was consistently lower for all additives at 60 °C compared to 100 °C, it can be inferred that the tribofilm formed through chemisorption of ester fragments on the contact surface contributes to reduced friction.

To obtain deeper insight into the tribofilm’s chemical composition, NEXAFS analysis was conducted alongside ToF-SIMS. Figure 12 presents the C K-edge spectra collected from inside and outside the wear tracks on plates tested with mixed GO, triolein, and oleic acid at 60 °C. For the GMO sample, a wear track was not visibly apparent in the instrument, so NEXAFS analysis could not be performed. The spectra exhibited nearly identical features across all samples, with a noticeable peak at 288.5 eV that could be attributed to the π* resonance of the carbonyl C=O bond, indicating the presence of an ester or acid functionality both inside and outside the wear tracks.^50^ Additionally, a smaller peak near 290.3 eV, previously associated with carboxylic acid (COOH) groups,^51,52^ was detected exclusively inside the wear tracks. This observation suggests the formation of oleate ions within the wear tracks, likely resulting from the hydrolysis of GMO and the thermal decomposition of GDO and triolein at lower temperatures.

This observation suggests the formation of oleate ions within the wear tracks, likely resulting from the hydrolysis of GMO and the thermal decomposition of GDO and triolein at lower temperatures.

These findings align well with the ToF-SIMS results, which revealed a distinct fingerprint peak at *m*/*z* 281.2, attributed to the oleate ion ([C_18_H_33_O_2_]^−^), across all additives at 60 °C. While the NEXAFS data are preliminary and need further analysis, the combined evidence supports the conclusion that chemisorption plays a crucial role in tribofilm formation and contributes significantly to friction reduction at lower temperatures.

**Figure 12:**
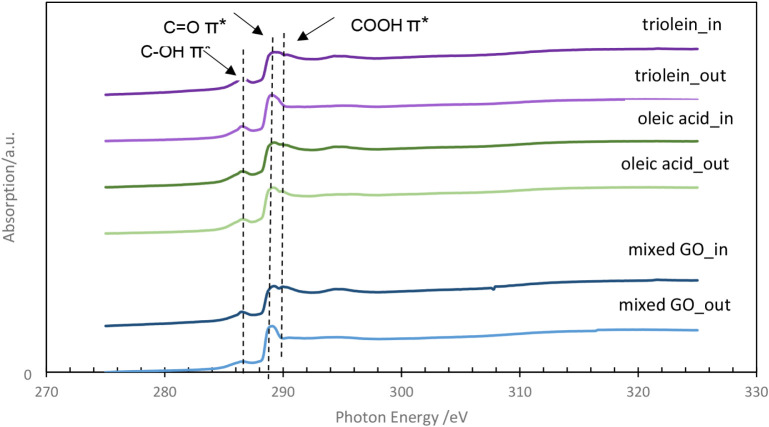
C K-edge spectra obtained from inside and outside the wear tracks on plates tested with mixed GO, triolein, and oleic acid at 60 °C. In and out mean inside and outside the wear track, respectively.

## 6 Conclusions

To minimize the environmental impact of lubricant additives while enhancing tribological efficiency, this study investigated the effects of lubricant combinations, temperature, and reciprocating motion on film chemistry and friction behavior. For the first time, the strong synergistic effect between three different OFMs has been reported. This lubricant combination demonstrated a 35% friction reduction at a temperature of 60 °C compared to the base oil under severe reciprocating conditions. This combination also exhibited the lowest boundary friction coefficient at a temperature of 60 °C among the tested additives, which is associated with the formation of a thicker tribofilm at the contact interface, as confirmed by the HRTEM image.

The difference in lubrication efficiency of these additives at 60 and 100 °C is attributed to the physical and chemical properties of the formed tribofilms. Presence of oleate fragments in the tribofilm formed at 60 °C, as evidenced by ToF-SIMS and NEXAFS analyses, indicates the formation of a robust oleate tribofilm, highlighting the crucial role of chemisorption in friction reduction at this temperature. The wear volume and wear scar width measurements indicated a significant improvement in wear performance, especially at a lower temperature, suggesting the presence of a more effective mechanical barrier composed of friction modifiers.

Moreover, the results underscore the crucial role of reciprocating motion in tribofilm development and stability. While sliding facilitates the mechanochemical activation and decomposition of additives at lower temperatures, promoting the chemisorption of tribofilm, it may also accelerate film removal under higher thermal and mechanical stress, thereby reducing tribofilm effectiveness.

Overall, this work highlights the importance of molecular structure, temperature effects, mechanical action, and additive combinations in developing environmentally friendly and high-performance organic friction modifiers.
